# Highly Efficient Multi-Step Oxidation Bioanode Using Microfluidic Channels

**DOI:** 10.3390/ijms222413503

**Published:** 2021-12-16

**Authors:** Tomohiro Komatsu, Kazuki Hishii, Michiko Kimura, Satoshi Amaya, Hiroaki Sakamoto, Eiichiro Takamura, Takenori Satomura, Shin-ichiro Suye

**Affiliations:** 1 Department of Advanced Interdisciplinary Science and Technology, Graduate School of Engineering, University of Fukui, Fukui 910-8507, Japan; tomohirok89@gmail.com; 2 Department of Frontier Fiber Technology and Science, Graduate School of Engineering, University of Fukui, Fukui 910-8507, Japan; hishiik@biofiber-fukui.com (K.H.); kimuram@biofiber-fukui.com (M.K.); e_takamr@u-fukui.ac.jp (E.T.); suyeb10@u-fukui.ac.jp (S.-i.S.); 3 Department of Mechanical Engineering, The University of Tokyo, 7-3-1, Hongo, Bunkyo-ku, Tokyo 113-8656, Japan; amaya@mesl.t.u-tokyo.ac.jp; 4 Department of Applied Chemistry and Biotechnology, Graduate School of Engineering, University of Fukui, Fukui 910-8507, Japan; satomura@u-fukui.ac.jp

**Keywords:** biofuel cells, bioanode, multi-step cascade reactions, efficient electron transfer, microfluidic system, immobilized enzymes method

## Abstract

With the rapid decline of fossil fuels, various types of biofuel cells (BFCs) are being developed as an alternative energy source. BFCs based on multi-enzyme cascade reactions are utilized to extract more electrons from substrates. Thus, more power density is obtained from a single molucule of substrate. In the present study, a bioanode that could extract six electrons from a single molecule of L-proline via a three-enzyme cascade reaction was developed and investigated for its possible use in BFCs. These enzymes were immobilized on the electrode to ensure highly efficient electron transfer. Then, oriented immobilization of enzymes was achieved using two types of self-assembled monolayers (SAMs). In addition, a microfluidic system was incorporated to achieve efficient electron transfer. The microfluidic system, in which the electrodes were arranged in a tooth-shaped comb, allowed for substrates to be supplied continuously to the cascade, which resulted in smooth electron transfer. Finally, we developed a high-performance bioanode which resulted in the accumulation of higher current density compared to that of a gold disc electrode (205.8 μA cm^−2^: approximately 187 times higher). This presents an opportunity for using the bioanode to develop high-performance BFCs in the future.

## Highlights

- Development of microfluidic electrode system.
- Efficient electron transfer in a multi-step cascade reaction.
- Tetra-enzyme immobilized electrode.
- Eight-fold increase in current density.

## 1. Introduction

The use of fossil fuels as an energy source in our daily lives has been experiencing a decline over the past several decades, which is cause for great concern. The burning of fossil fuels, however, leads to the production of carbon dioxide, sulfur oxide, and nitric oxide, which contributes significantly to global warming. Environmentally friendly energy sources, such as solar energy, wind energy, and hydraulic power, have been in high demand to limit the advancement of global warming. However, these energy sources also have drawbacks, such as being dependent on the location and the weather.

Recently, there has been an interest in the use of biomass and fuel cells as alternative energy sources. In general, biomass is a renewable organic energy source obtained from animal or plant material, while fuel cells use inorganic matter such as oxygen and hydrogen. However, due to the requirements of high temperatures and pressures during operation, this could be quite dangerous. Taking safety into consideration, biofuel cells (BFCs), which use organic substances as fuel, have been developed for practical use.

BFCs utilize organic compounds as fuel to generate protons at the anode (bioanode). Electricity is then generated when oxygen is converted to water at the cathode (biocathode). BFCs have several advantages, which include mild operation conditions, low safety risk, and the use of renewable energy sources. BFCs also have several drawbacks. First, glucose is generally used as fuel in BFCs, which can be obtained from starch [1]. This implies that a competing interest might emerge in using glucose for either fuel or food. Sarbon reported on this issue and suggested using gelatin instead of glucose. Components in gelatin include hydroxyproline, glycine, alanine, and proline, which might be considered as food waste [2]. His work shows that gelatin may also be used as fuel for BFCs. Second, only two electrons are extracted from one molecule of glucose in general BFCs, which might affect the output of BFCs. Several investigations of multi-enzyme systems have been carried out to improve electron extraction [3,4,5,6]. Matsumoto reported a two-step cascade reaction between glucose and 5-ketogluconate [7]. In Step 1 (the reaction from glucose to gluconate), the electron is transported to a reduced form of nicotinamide adenine dinucleotide (NADH) at the coenzyme binding site of glucose dehydrogenase. Then, the electron is transported through the enzyme diaphorase, which catalyzes the dehydrogenation reaction that converts NADH to NAD^+^. Finally, electrons are received by the electrode surface via a mediator in Step 2 (reaction from gluconate to 5-ketogluconate). Each reaction results in the extraction of two electrons from one molecule of substrate. Therefore, four electrons are obtained in this cascade, and as a result, the power density obtained during this cascade reaction is 1.6 times higher than that of a single reaction of glucose to gluconate (Step 1).

Finally, the last drawback is that the output of BFCs is dependent on the enzymatic activity on the electrode [8]. In general, the output of BFCs is higher if the enzymatic activity of the enzyme which helps the redox reaction is higher. However, reacting efficiently is difficult on the electrode because the substrate binding site of the enzyme could hide via the ordinary immobilization way of the enzyme. Thus, the substrate could not bind to the substrate binding site efficiently. Several investigations have used various enzyme immobilization methods to promote efficient electron transfer between the enzyme and the electrode surface [9,10,11,12,13,14,15]. Kim reported on the immobilization of enzymes through electrostatic bonds for the development of a glucose biosensor [16]. The surface modification led to an improvement of the enzymatic activity on the electrode. As a result, the oxidation peak current (*I*_pa_) was twice as high as that obtained when using covalent bonding. In addition to using electrostatic bonding, the orientation of the enzymes on the electrode has also been investigated as an enzyme immobilization method. For this method, the orientation of the enzyme is controlled to close the active site of the enzyme to the electrode surface, allowing electron transfer to occur efficiently.

**Figure 1:**
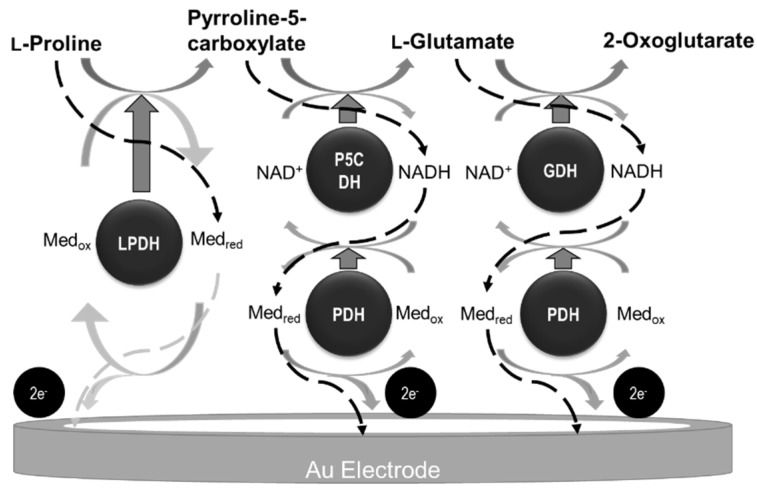
Scheme of cascade reaction in the present study.

In the present study, we aimed to construct a novel bioanode which has much higher current density combining methods of construction of BFC as mentioned above. Firstly, a bioanode capable of extracting six electrons from a single molecule of L-proline via a three-enzyme cascade reaction was developed and investigated for possible use in a BFC. Dye-linked L-proline dehydrogenase (LPDH), pyrroline-5-carboxylate dehydrogenase (P5CDH), and L-glutamate dehydrogenase (GDH) were co-immobilized on the electrode (Figure 1). Two dehydrogenases in the cascade reaction require NADH as a cofactor, which are also linked with NADH dehydrogenase for NADH regeneration and the transfer of electrons to the electrode. Therefore, proline dehydrogenase, which shows NADH dehydrogenase activity (PDH), is also utilized to extract electrons. In general, enzyme reactions are not smooth when several enzymes are on the electrode [17]. Therefore, these enzymes were immobilized on the electrode for highly efficient electron transfer. Then, the orientations of the immobilized enzymes were controlled through the use of self-assembled monolayers (SAM). Finally, we were able to confirm that electron transfer took place in this cascade reaction, and the optimum molar ratio of enzymes and SAM were investigated and evaluated to obtain a high current density.

**Figure 2:**
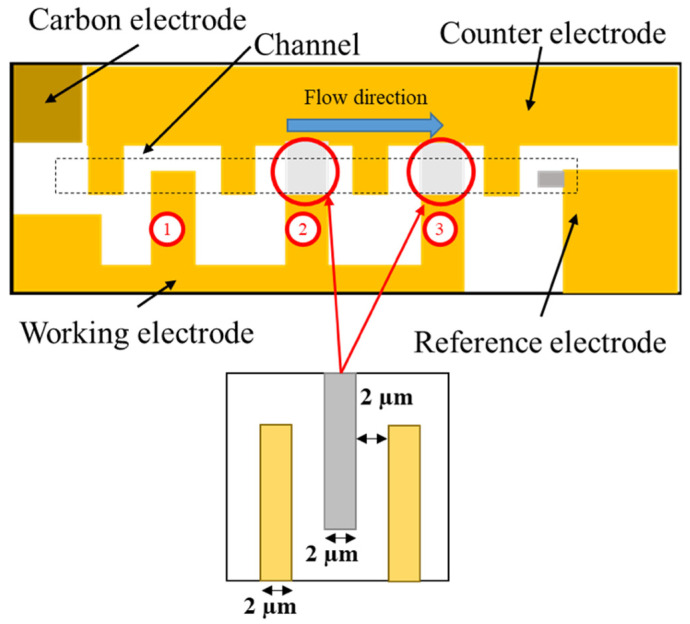
Illustration of an electrode with an incorporated microfluidic system. The figure below shows the detailed structure of the comb electrodes of the second and third electrodes.

**Figure 3:**
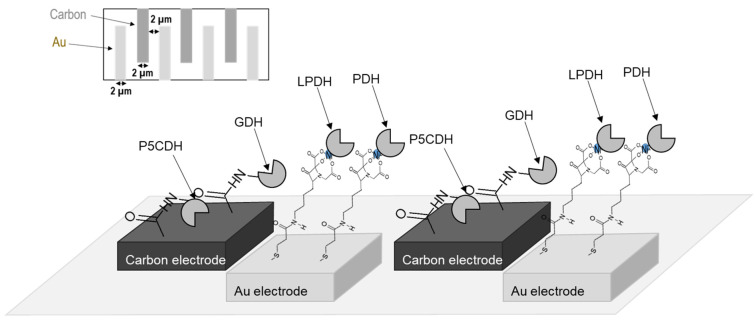
Illustration of the arrangement of enzymes on the microfluidic system electrodes in this study. The upper left figure shows the detailed structure of the comb electrodes of the second and third electrodes.

Moreover, the analytical methods of samples in flow such as flow injection analysis (FIA) and micro total analysis systems (µTAS) were developing in recent years [18,19]. To improve the electron transfer efficiency, an electrode with an incorporated microfluidic system was developed (Figure 2). The gold and carbon electrodes were arranged in a tooth-shaped comb. This arrangement would allow for more efficient electron transfer by keeping the distance between the carbon and gold electrodes constant (Figure 3).

Furthermore, the supply of substrate (L-proline) was stable when using a microfluidic system. Finally, we have confirmed that electron transfer took place in this cascade using a microfluidic system, and that the current density was much higher than that of the disc electrode.

## 2. Results and Discussion

### 2.1. Confirmation of Electron Transfer during the Tetra-Enzymatic Cascade Reaction on the Gold Disc Electrode

**Figure 4:**
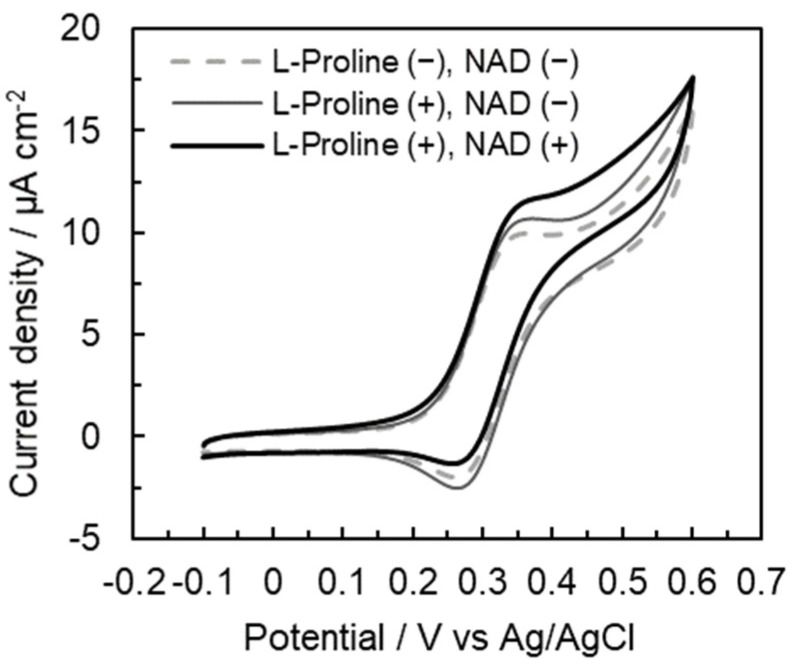
CV grams of the tetra-enzyme immobilized electrode using L-proline or L-glutamate as the substrate.

To confirm the increase in electron transfer on the tetra-enzyme immobilized electrode, an electrochemical evaluation of each step was carried out. We observed a pair of redox peaks derived from the redox coupling of Fe(III)/Fe(II) ions in ferrocenecarboxylic acid at approximately 0.35 V in each voltammogram (Figure 4). First, the oxidation current increased in the presence of L-proline. Furthermore, the oxidation peak increased even more in the presence of L-proline and NAD^+^. In these results, we confirmed that this three-step cascade reaction was proceeded in the presence of the substrate (L-proline) and coenzyme (NAD^+^). Thus, the electron was trasfered in this three-step cascade as in Figure 1.

### 2.2. Comparison of Electrochemical Characterization of the Microfluidic System Electrode with That of Gold Disc Electrode Using Tetra-Enzyme Cascade

**Figure 5:**
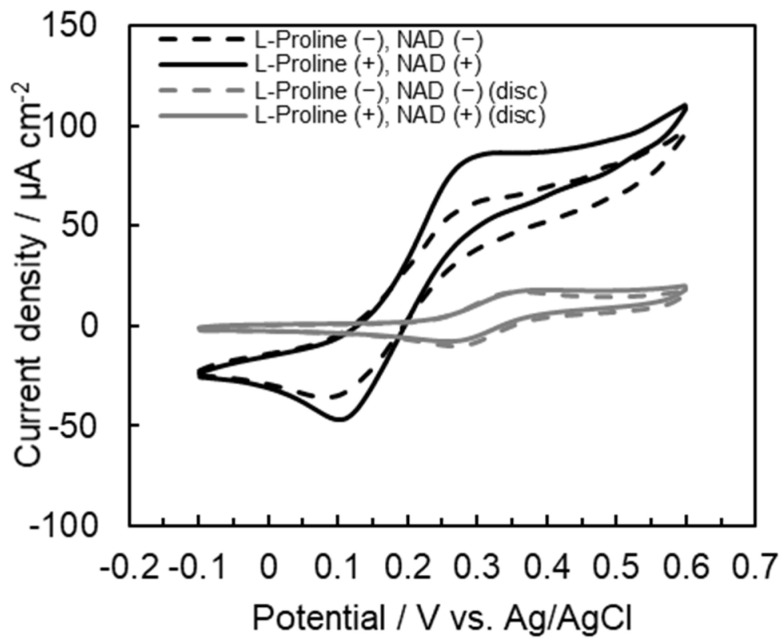
CV grams of the tetra-enzyme immobilized electrode: comparison of the microfluidic system electrode and the disc electrode.

In the microfluidic electrode system, electron transfer in this three-step cascade reaction was also observed (Figure 5). Using this electrode, the current density was 23.2 µA cm^−2^, which was 21 times higher than that obtained using a disc electrode (Table 1). In this result, the continuous reaction in the three-step using mucrofluidic electrode proceeded more smoothly than that using gold disc electrode because the separate arrangement of the immobilized electrodes in the three stages made the enzymes arrange efficiency.

### 2.3. Evaluation of Electrochemical Characteristic Differences between Flow of Substrate and No Flow of Substrate

**Figure 6:**
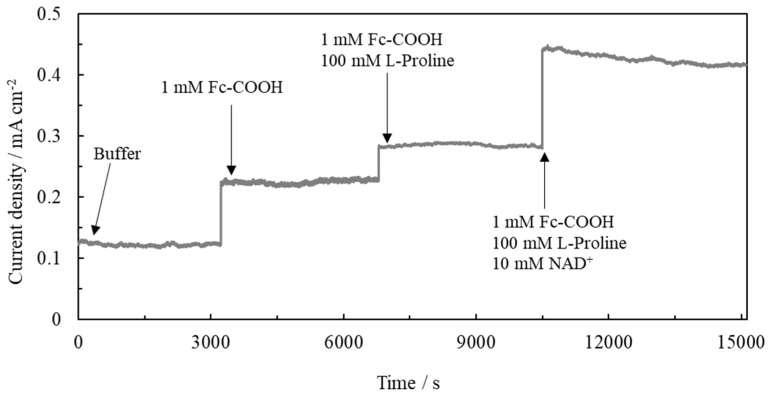
Current density in tetra-enzyme immobilized electrode using a microfluidic system.

We investigated the electrochemical behavior under continuous substrate supply in a flow channel system in the tetra-enzyme immobilized electrode. The results show that the current density increased after applying the substrate and coenzyme, respectively. Thus, the cascade reaction in this microfluidic system was confirmed (Figure 6). In addition, the current density was 205.8 μA cm^−2^ after adding 100 mM L-proline and 10 mM NAD^+^. It was approximately eight times higher compared to that of this electrode without a continuous flow. These results indicate that electron transfer in this cascade was more efficient using the microfluidic system. This was thought to be because the continuous reaction proceeded smoothly due to the separate arrangement of the immobilized electrodes in the three stages, and also because the diffusion of substrates, enzyme reaction products, and mediators was improved due to continuous supply of them in this flow type of the pump, thus achieving an efficient multi-enzyme reaction.

## 3. Materials and Methods

### 3.1. Materials

L-Proline (substrate) and ferrocenecarboxylic acid (mediator) that were used for electrochemical measurements were purchased from Wako (Osaka, Japan). β-Nicotinamide adenine dinucleotide (β-NAD^+^), oxidized form of co-enzyme, was purchased from Oriental Yeast (Tokyo, Japan). The 3,3′-dithiobis[*N*-(5-amino-5-carboxypentyl)-propionamide-*N*’,*N*’-diacetic acid] dihydrochloride (C_2_-NTA) and dithiobis-succinimidyl undecanoate (DSU) were purchased from Dojindo Laboratories (Kumamoto, Japan). All chemicals that were used during this study were of analytical grade. Cyclic voltammetry (CV), amperometry, and linear sweep voltammetry experiments were performed using a Model 800 B electrochemical analyzer (BAS Inc., Tokyo, Japan).

### 3.2. Methods

#### 3.2.1. Purification of LPDH

LPDH was expressed in *Escherichia coli* BL21-Codonplus (DE3)-RIL competent cells, using a pET15b/Ape_1267.1 expression vector containing the LPDH gene from the hyperthermophilic archaeon *Aeropyrum pernix*. *E. coli* transformed with the pET15b expression vector were cultured at 37 °C in LB medium containing ampicillin (50 µg/mL) until the OD_600_ reached 0.6. Expression was induced by the addition of 1 M isopropyl-β-D-thiogalactopyranoside (IPTG). Cultivation was allowed to continue for 4 h at 37 °C. The cells were collected by centrifugation (10,000 rpm for 10 min at 4 °C) and resuspended in 10 mM sodium phosphate buffer (pH 7.2) containing 0.1 M NaCl. The crude extract was prepared by ultrasonication, and heated to 70 °C, which was maintained for 10 min. Finally, His-tagged LPDH was purified using Ni-NTA chromatography with a HisTrap HP column.

#### 3.2.2. Purification of P5CDH

P5CDH was expressed in *E. coli* BL21-Codonplus (DE3)-RIL competent cells, using a pET11a/TT0033 expression vector containing the P5CDH gene from the hyperthermophilic archaeon *Thermus thermophilus*. *E. coli* transformed with the pET11a expression vector was cultured at 37 °C in SB medium containing ampicillin (50 µg/mL) until the OD_600_ reached 0.6. Expression was induced by the addition of 1 M IPTG. Cultivation and cell collection were performed in the same manner as described in Section 3.2.1. Subsequently, the cells were resuspended in 20 mM Tris-HCl buffer (pH 8.0) containing 5 mM 2-mercaptoethanol and 0.5 M NaCl. The crude extract was prepared by ultrasonication and heated at 70 °C and maintained for 13 min. Finally, P5CDH was purified using a Q Sepharose column.

#### 3.2.3. Purification of GDH

GDH was expressed in *E. coli* BL21-Codonplus (DE3)-RIL competent cells using a pET11a/Pise_1816 expression vector containing the GDH gene from the hyperthermophilic archaeon *Pyrobaculum islandicum*. *E. coli* transformed with the pET11a expression vector was cultured at 37 °C in SB medium containing ampicillin (50 µg/mL) until the OD_600_ reached 0.6. Expression was induced by the addition of 1 M IPTG. Cultivation and cell collection were performed in the same manner as described in Section 3.2.1. Subsequently, the cells were resuspended in 10 mM potassium phosphate buffer (pH 7.2) containing 10% (*v*/*v*) glycerol, 1 mM EDTA (ethylenediaminetetraacetic acid), and 0.1 mM DTT (dithiothreitol). The crude extract was prepared by ultrasonication and heated at 85 °C and maintained for 10 min. Finally, GDH was purified using a Red Sepharose CL-4B column.

#### 3.2.4. Purification of Proline/NADH Dehydrogenase (PDH)

PDH was expressed in *E. coli* BL21-Codonplus (DE3)-RIL competent cells, using a pET15b/PH1748, OH1749, PH1750, and PH1751 expression vectors containing the PDH/NADHDH gene from the hyperthermophilic archaeon *Pyrococcus horokoshii*. *E. coli* transformed with the pET15b expression vector was cultured at 37 °C in SB medium containing ampicillin (50 µg/mL) until the OD_600_ reached 0.6. Expression was induced by the addition of 1 M IPTG. Cultivation and cell collection were performed in the same manner as described in Section 3.2.1. Moreover, the cells were resuspended in 10 mM potassium phosphate buffer (pH 7.0) containing 0.1 M NaCl. The crude extract was prepared by ultrasonication and heated at 70 °C and maintained for 10 min. Finally, His-tagged PDH was purified using Ni-NTA chromatography with a HisTrap HP column.

#### 3.2.5. Fabrication of Microfluidic Channel

**Figure 7:**
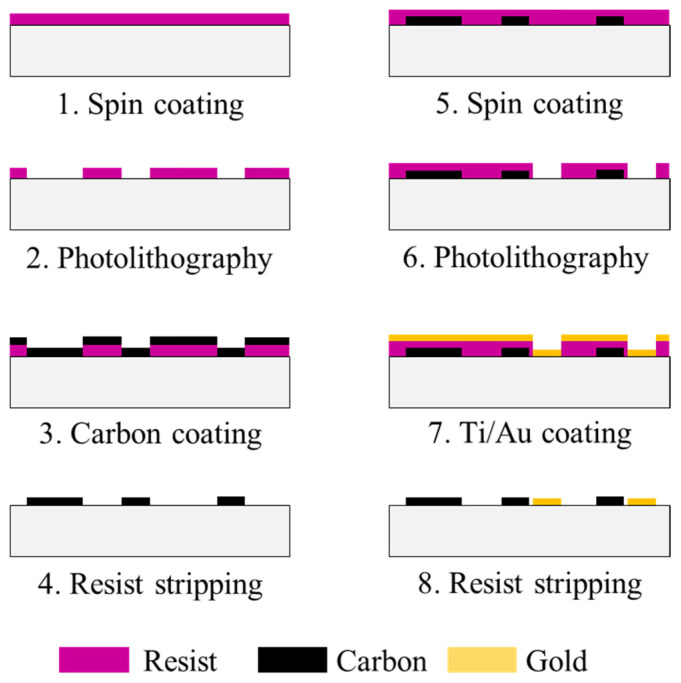
Schematic illustration of device design and interdigitated microelectrodes.

The microfluidic device with interdigitated microelectrodes for BFC was fabricated using UV lithography and lift-off process on a glass substrate (Matsunami Glass Ind., Ltd., Osaka, Japan) with 26 mm width, 1 mm thickness, and 76 mm length. Figure 7 shows the fabrication procedure of the interdigitated microelectrodes for BFC. (1) First, a photoresist (AZ1500, AZ Electronic Materials) was spin-coated on the substrate and baked. (2) Then, UV light (405 nm) was applied (90 mJ/cm^2^) by a maskless lithography system (D-light DL-1000, Nano System Solutions) to make the patterns of the electrodes, interdigitated microelectrodes and the photoresist were developed. (3) Next, 100 nm carbon film layers were deposited by sputtering. (4) The patterns of electrodes and interdigitated microelectrodes were formed by lifting off the photoresist and the carbon in acetone. (5) Afterwards, a photoresist was spin-coated on the substrate and baked again. (6) UV light was applied to make the patterns of the electrodes; interdigitated microelectrodes and the photoresist were developed. (7) Next, 10 nm Ti film and 100 nm Au film layers were deposited by electron beam evaporation. (8) The patterns of electrodes and interdigitated microelectrodes were formed by lifting off the photoresist and the metals in acetone. Finally, a polydimethylsiloxane microchannel (width 2 mm, height 500 μm) was attached onto the substrate.

#### 3.2.6. Preparation of Tetra-Enzyme Immobilized Electrode

A pretreated Au disc electrode (diameter: 0.3 mm) was immersed in mixed SAM containing C_2_-NTA and DSU at room temperature (25 °C) for 3 h. Subsequently, P5CDH and GDH were dropped onto the electrode and placed on at room temperature for 30 min. The electrode was then immersed in 100 mM NiSO_4_ at room temperature for 10 min. Finally, LPDH and PDH were dropped onto the electrode at room temperature for 30 min. These enzymes were prepared with the same molecular number.

For the microfluidic electrode, which contains both carbon and gold electrodes, a carboxyl group was introduced onto the carbon electrode via electrochemical oxidation in 2.5% potassium dichromate and 10% nitric acid solution. A succinimide group was then introduced onto the carbon electrode. In addition, C_2_-NTA was dropped onto the gold electrode at room temperature for 3 h. Finally, P5CDH and GDH were dropped and immobilized on the carbon electrode through the *N*-hydroxysuccinimidyl group, and LPDH and PDH with a His-tag were dropped and immobilized with high orientation on the gold electrode via C_2_-NTA in the same manner as described above.

#### 3.2.7. Electrochemical Characterization of the Tetra-Enzyme Immobilized Electrode

Electrochemical characterization of the tetra-enzyme immobilized electrode with ferrocenecarboxylic acid was performed by CV and chronoamperometry (CA). CV was conducted from −0.1 to 0.6 V at 10 mV/s at 50 °C. The electrolyte solution was 50 mM Tris-HCl buffer (pH 8.0), while the concentrations of ferrocenecarboxylic acid, L-proline, and NAD^+^ were 0.1, 100, and 0.1 mM, respectively. CA was conducted at +0.5 V under a flow rate of 1.0 mL/min at 50 °C (using Eyela micro tube pump, Tokyo Rikakikai Co., Ltd., Tokyo, Japan). The electrolyte solution was 50 mM Tris-HCl buffer (pH 8.0), while the concentrations of ferrocenecarboxylic acid, L-proline, and NAD^+^ were 1, 100, and 10 mM, respectively.

## 4. Conclusions

In this study, we confirmed that more electron transfer occurred in a three-step reaction on a tetra-enzyme-immobilized electrode. The results showed that the tetra-enzyme cascade reaction proceeded much more efficiently on the newly developed microfluidic electrode system than on the disc gold electrode. As a result, we obtained 23.2 μA cm^−2^, which was a 21-fold increase in current density with the microfluidic electrode system in comparison to that of the gold disc electrode. Furthermore, with the implementation of a continuous flow channel system, we were also able to observe the electrochemical properties of this microfluidic electrode system. We observed that there was an 8.87-fold increase in current density (205.8 μA cm^−2^) after applying the redox agent, substrate, and coenzyme, respectively, in this continuous flow system in comparison when no flow was present. Finally, we have constructed a new bioanode which improved current density using a three-step cascade reaction, immobilized method of enzymes, and microfluidic system, and obtained almost 200-fold higher current density than that when using a gold disc electrode. Unfortunately, the current density obtained in this study still has issues for practical applications. However, improvement of this issue is expected by immobilization mediators on the electrode. Therefore, we believe that this bioanode can be utilized in BFCs is the first step for use in practical application in the future.

## Figures and Tables

**Table 1:** Comparison of microfluidic electrode with disc electrode.

Electrode	Δ Current Density (*I*_pa_) µA cm^−2^
Disc electrode	1.1
Microfluidic electrode	23.2
